# Synthesis of vacancy-rich titania particles suitable for the additive manufacturing of ceramics

**DOI:** 10.1038/s41598-022-19824-y

**Published:** 2022-09-14

**Authors:** Jaime A. Benavides-Guerrero, Luis Felipe Gerlein, Charles Trudeau, Debika Banerjee, Xiaohang Guo, Sylvain G. Cloutier

**Affiliations:** Department of Electrical Engineering, Ecole de Technologie Superieure, 1100 Notre-Dame West, Montreal, QC H3C 1K3 Canada

**Keywords:** Chemistry, Materials chemistry

## Abstract

In the last decades, titania (or TiO_2_) particles played a crucial role in the development of photo-catalysis and better environmentally-friendly energy-harvesting techniques. In this work, we engineer a new generation of TiO_2_ particles rich in oxygen vacancies using a modified sol–gel synthesis. By design, these vacancy-rich particles efficiently absorb visible light to allow carefully-controlled light-induced conversion to the anatase or rutile crystalline phases. FTIR and micro-Raman spectroscopy reveal the formation of oxygen vacancies during conversion and explain this unique laser-assisted crystallization mechanism. We achieve low-energy laser-assisted crystallization in ambient environment using a modified filament 3D printer equipped with a low-power laser printhead. Since the established high-temperature treatment necessary to convert to crystalline TiO_2_ is ill-suited to additive manufacturing platforms, this work removes a major fundamental hurdle and opens whole new vistas of possibilities towards the additive manufacturing of ceramics, including carefully-engineered crystalline TiO_2_ substrates with potential applications for new and better photo-catalysis, fuel cells and energy-harvesting technologies.

## Introduction

Thanks to their unique properties, titanium dioxide (TiO_2_) or *titania* particles have generated a tremendous interest from the scientific community in the last decades^1–6^. Today, they play an essential role in multiple applications ranging from photo-catalysis to energy-harvesting^7,8^. Their chemical stability, nontoxicity, large bandgap, oxidizing power and photo-catalytic properties all strongly depend on their crystalline structure^1,9,10^. Conventional synthesis routes include mechanically-induced self-sustaining reactions^11^, direct oxidation of titanium via chemical or physical vapor depositions^12^, micro-emulsion methods^13^, hydrothermal or solvothermal^14^ methods, spray- or laser-pyrolysis^15,16^, and sol–gel chemistry^17–19^. The sol–gel chemistry constitutes a widely-popular environmentally-friendly synthesis route, with deep roots in the so-called *green-* or *soft-chemistry*^20,21^. It can also allow a careful control of the particles sizes at the nanoscale level, potentially triggering new quantum confinement-specific properties^17–19,22^. In TiO_2_, this regime is extremely difficult to reach due to a Bohr radius under 2.4 nm^23^. Fortunately, these properties also be significantly and controllably altered using various defects or impurities^24,25^. For example, surface defects including oxygen vacancies can also dramatically affect the structural, physical and chemical properties of TiO_2_ particles^26,27^. For instance, the presence of surface defects in metal-oxides can allow anions and cations to assume a variety of charged surface states^28,29^. This phenomena leads to multiple applications including photocatalysis^30–32^, corrosion protection^33^, sensors^34^, microelectronics^35^, magnetic recording devices^36,37^ and microporous materials^38^.

Surface defects or vacancies can also modify the electronic levels^39^, optical absorption and emission properties and bring-in new roperties^40^. Oxygen vacancies present the lowest energy formation among the surface defects^41^ which makes them ideal candidates to tailor the properties of oxide semiconductors such as TiO_2_^42^. In fact, nanostructured TiO_2_ boosts the formation of oxygen vacancies as a result of its higher active surface area^42^. Vacancies appear when there is an atom or ion missing in the lattice structure, resulting in cation or anion vacancies^43^. Cation vacancies occur when a positive ion is removed from its niche^38^. They create localized energy levels above the valence band maximum^44^. In contrast, anion vacancies appear when a negative ion is removed from its niche^38^. They create in-gap localized energy levels near the conduction band minimum^45^. Oxygen vacancies increase the visible light absorption by generating inter sub-band energy states so photons with less energies than the TiO_2_ band gap can be absorbed^44,46,47^. Moreover, It is known that the presence of oxygen vacancies and/or the Ti^3+^ oxygen vacancy associates in the TiO_2_ changes the color of the material from withe to yellow, blue, black or red^48,49^.

In this work, we establish how carefully-controlled hydrolytic TiO_2_ sol–gel chemistry can provide a precise control over the incorporation of oxygen vacancies at the particles’ interface. In turn, these dark-colored oxygen vacancy-rich amorphous TiO_2_ particles absorb very efficiently visible light. The increase in oxygen vacancies potentially leads to increase their photo-catalysis and energy-harvesting performances^39,50–54^. Indeed, we successfully achieve room-temperature conversion to anatase or rutile TiO_2_ in air using only a low-power laser. Since the high-temperatures required to crystallize TiO_2_ are detrimental for additive manufacturing platforms, this last breakthrough opens new vistas of possibilities towards the additive manufacturing of ceramics and the design of engineered crystalline TiO_2_ substrates for new and better photo-catalysis, fuel cells and other energy-harvesting technologies.

### Synthesis

**Figure 1:**
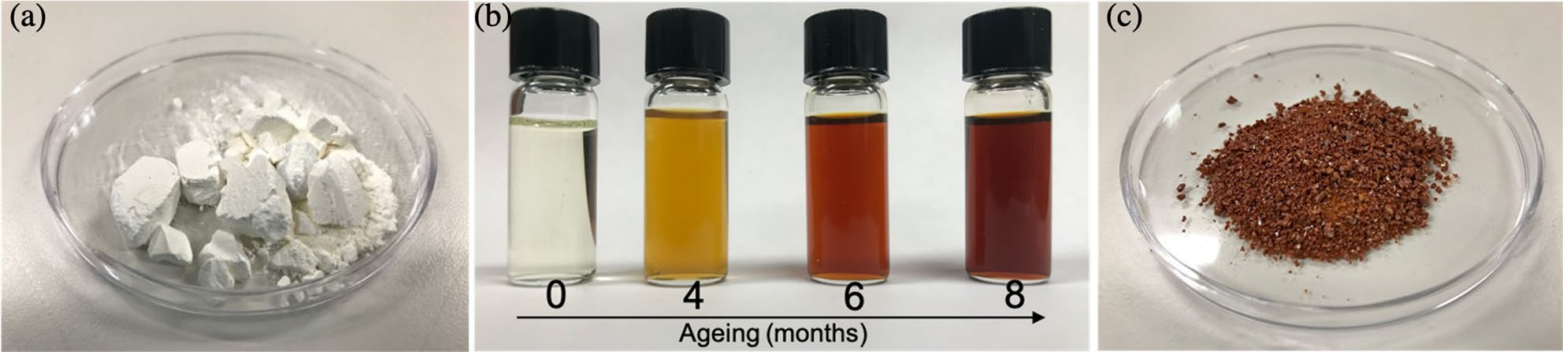
Amorphous TiO_2_ nanoparticles obtained by sol–gel technique. (**a**) White TiO_2_ powder synthesized trough a standard sol–gel reaction. (**b**) Ageing of the solution used to produce oxygen vacancy-rich (red) amorphous TiO_2_ nanoparticles. (**c**) Oxygen vacancy-rich (red) amorphous TiO_2_ powder obtained after solvent evaporation.

As a control experiment, we use a common sol–gel synthesis of amorphous TiO_2_ through the hydrolysis and condensation of a metal alkoxides^55^. In this case, we use titanium tetrabutoxide Ti(OBu)_4_ as the precursor^56^. In this relatively standard synthesis, the hydrolysis reaction is exothermic and occurs in a matter of seconds^56^. It is followed by the condensation reaction, where the white amorphous TiO_2_ nanoparticle aggregate is formed and precipitates to the bottom of the beaker^56^. While the whole process is completed in a few seconds, it is common practice to age the system for up-to 72 h^57^ before evaporating the solvent and recuperate a white amorphous TiO_2_ powder as shown in Fig. 1a.

To engineer the titania particles’ properties, we begin by slowing-down the reaction kinetics in an attempt to synthesize amorphous TiO_2_ material rich in oxygen vacancies. Most common alkoxy groups used in the TiO_2_ synthesis contain between two carbon atoms (ethoxy) and four carbon atoms (butoxy). Their reactivity towards hydrolysis decreases as the number of carbon atoms in the chain increases^58^. To decrease their reactivity towards water, it is common to dilute the nanoparticles in alcohols or mix with complexing agents^58^. In our case, acetylacetone (acac) is used as a complexing agent in order to chelate the metallic cation on the Ti(OBu)_4_. The reaction between acac and Ti(OBu)_4_ occurs trough an interexchange substitution mechanism and it can be represented as follows^58^:

$${\text{Ti}}\left( {{\text{OBu}}^{{\text{n}}} } \right)_{{4}} + {\text{ acacH}}\rightarrow {\text{Ti}}\left( {{\text{OBu}}^{{\text{n}}} } \right)_{{{4} - {\text{x}}}} + {\text{ xBuOH}}$$

As such, the standard hydrolysis and condensation reactions participating in the conventional sol–gel process can be controlled through the hydrolysis and complexation molar ratios. We define these two ratios as r_c_ = [acac]/[Ti] and r_w_ = [H_2_O]/[Ti], respectively. These ratios can be varied in order to obtain precipitates, opaque gels, transparent gels, stable sols and cluster solutions^58^. To obtain transparent gels, the values we use for the hydrolysis and complexation ratios are summarized in the Table 1.

**Table 1:** Hydrolysis and complexation ratios used for the synthesis of the standard (white) and the oxygen vacancy-rich (red) amorphous TiO_2_ particles.

TiO_2_ powder	r_w_ = [H_2_O]/[Ti]	r_c_ = [acac]/[Ti]
White TiO_2_ (Fig. 1a)	1.46	0
Red TiO_2_ (Fig. 1b)	1.46	1.96

We rapidly observe that the complexation ratio significantly affects the coloration of the TiO_2_ sol–gel system. While a pale-yellowish color is observed using the standard synthesis (r_c_ = 0), the system presents an intense yellow color when the acetylacetone (acac) concentration is increased (r_c_ = 1.96). As shown in Fig. 1b, its color also continues to gradually evolve from yellow to blood-red in a very slow aging process during up-to 8 months. At this time, a burnt-red TiO_2_ powder shown in Fig. 1c can be obtained after solvent evaporation. This drastic change in color can be directly attributed to the formation of surface defects or oxygen vacancies and it originates from the charge transfer from the chelant agent (acac) to the Ti^4+^ ion^59^. Titanium precursors such as ethoxyde Ti(OC_2_H_5_)_4_, isopropoxide Ti(OC_3_H_7_)_4_ and in our case, butoxide Ti(OC_4_H_9_)_4_ tend to form peroxo complexes presenting an intense orange color in solution^58^. These, peroxo groups are also known to significantly enhance the visible light photo-excitation of TiO_2_^60–63^.

## Results and discussions

### Synthesis and properties

**Figure 2:**
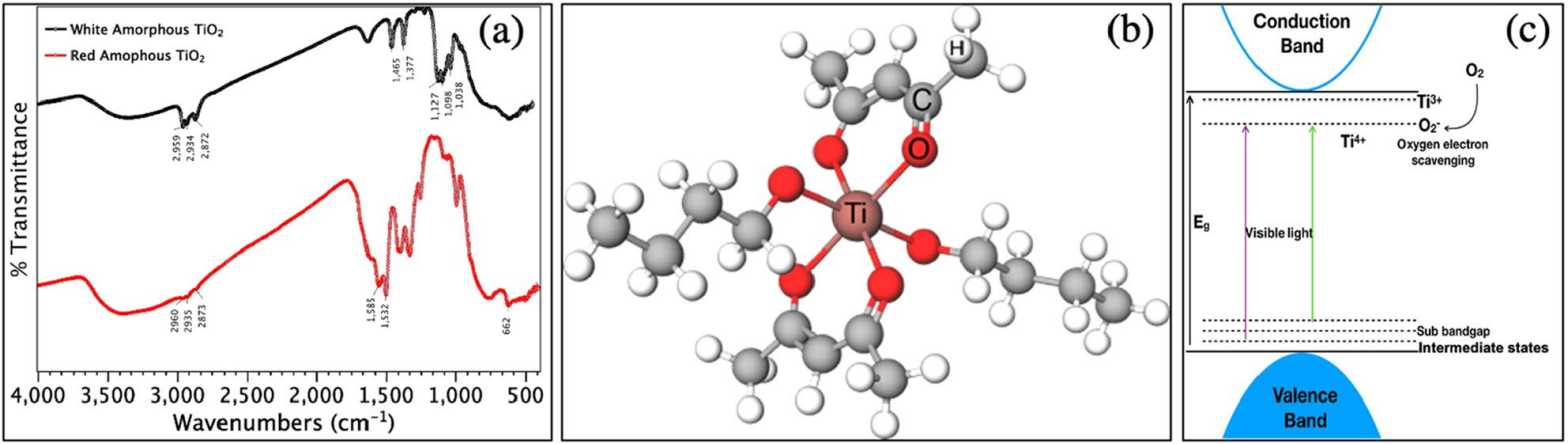
Oxygen vacancy-rich (red) amorphous TiO_2_, structure and properties. (**a**) Comparison of the FTIR absorption spectra for the standard (white) and the vacancy-rich (red) amorphous TiO_2_. (**b**) Schematic representation of the Ti(OBu)_4_ molecule chelated by acetylacetone (acac) during the synthesis. (**c**) Schematic representation of the energy diagram with sub-bandgap states due to oxygen vacancies, explaining the dark color. The proposed reaction for the laser-assisted phase transition process is also illustrated.

In the Fig. 2a, it is possible to observe that the standard (white) amorphous TiO_2_ clearly shows bands around 1466 cm^−1^ and 1378 cm^−1^ attributed to the tension vibrational modes of the aliphatic groups –CH_2_ and –CH_3_ from the Ti(OBu)_4_ and ethanol^64^. The bands around 1128 cm^−1^, 1099 cm^−1^ and 1039 cm^−1^ correspond to the vibrations of the Ti–O–C of the butoxy groups bonded directly to the titanium^64^. Interestingly, these bands (1128 cm^−1^ and 1039 cm^−^1) present a significant variation in their intensity ratios when both systems are compared. The intensity of the band at 1039 cm^-1^ is significantly reduced in the white amorphous TiO_2_ with more Ti (i.e. r_c_ = 0), while the intensity of the 1128 cm^−1^ band is much more pronounced in the red amorphous TiO_2_ synthesized with more acac (r_c_ = 1.96)^59^.

The bands at 2960 cm^−1^, 2935 cm^−1^ and 2873 cm^−1^ also contain information on the symmetric and asymmetric modes νCH_3_ and γCH_2_ for Ti(OBu)_4_ present in the white amorphous TiO_2_ and the Ti-acac complex present in the red amorphous TiO_2_^64,65^. These bands are sharper in the white amorphous TiO_2_ and less pronounced in the red amorphous TiO_2_, as a direct consequence of the chelation reaction between acac and Ti^64^. In contrast, the doublet at 1585 cm^−1^ and 1532 cm^−1^ observed exclusively in the red TiO_2_ corresponds to the vibrational modes νC = C and νC = O. The presence of this doublet is another direct consequence from the acac-Ti bonding^66^. Furthermore, the absence of the acac characteristic band at 1620 cm^−1^ suggests that it reacted completely by chelating the Ti cation^59,66^. The band at 662 cm^−1^ also appears exclusively in the red amorphous TiO_2_, and corresponds to the modes ν(C-CH3) and ν(Ti–O) of the aromatic ring formed between acac and Ti^65,67^. We can directly confirm from this FTIR analysis the formation of the Ti(OBu)_4_-acac complex, which reduces the chances of the condensation and polymerization reactions. Based on these FTIR observations, the cyclic dimeric structure shown in Fig. 2b can be proposed for the chelation process yielding the red amorphous TiO_2_ powder^68^.

This high concentration of oxygen vacancies creates intermediate states within the TiO_2_ bandgap as shown in Fig. 2c. These oxygen vacancies can yield very efficient light absorption, while augmenting the Urbach energy of the oxygen vacancy rich TiO_2_^69^. We confirmed the light absorption properties of the amorphous red TiO_2_ by UV–vis (Suppl. Figure S1). As such, we can now environ a light-assisted conversion process triggered at room temperature and under ambient conditions, having a vast supply of molecular oxygen available to participate in the process. In turn, this reaction facilitates the phase transition thanks to the ionic mobility created with oxygen vacancies^70^. In fact, oxygen molecules act as very efficient photo-excited electron scavengers, trapping the excited electrons from the conduction band into the surface states of the TiO_2_^71^. Then, the oxygen molecules are adsorbed at the surface of the red TiO_2_ nanoparticles to partially compensate these oxygen vacancies^72^. The presence of oxygen vacancies promotes the formation of Ti^3+^ sites in the crystal structure as the electrons left behind by the vacancy are distributed on neighboring Ti sites, reducing them^71,73,74^ from Ti^4+^ to Ti^3+^. Assisted by continuous irradiation, the adsorbed oxygen molecule passivates the TiO_2_ by bridging the metallic ions^75^. A schematic of this process is presented in Fig. 2c.

### Laser-induced crystallization of the oxygen vacancy-rich TiO_2_

**Figure 3:**
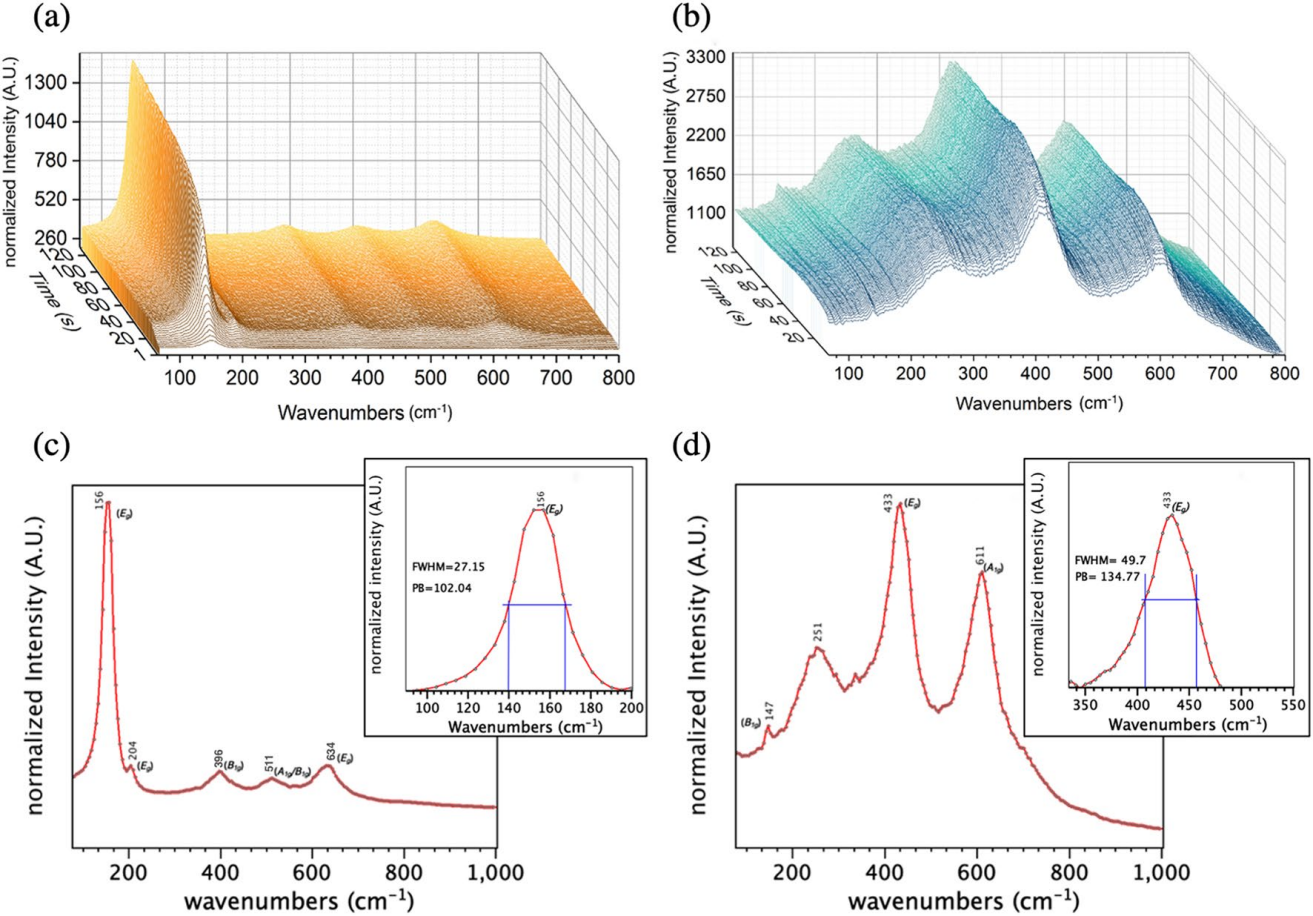
Transient Raman micro-spectroscopy measurements monitoring the laser-assisted conversion of the red amorphous TiO_2_. (**a**) Exposed to a 75 W/mm^2^ power density. (**b**) Exposed to a 445 W/mm^2^ power density. (**c**) Anatase and (**d**) rutile Raman spectra obtained using laser-assisted crystallization in ambient room environment. The insets show the E_g_ mode broadening for each obtained phase.

In this oxygen vacancy-rich (red) amorphous TiO_2_ we synthesize, the combined use of the oxygen vacancies and peroxo groups now offers the potential for laser-assisted crystallization. To demonstrate the rapid laser-assisted crystallization of amorphous red TiO_2_ powder, we use a Raman micro-spectroscopy system to record the transient evolution of the Raman signatures over a period of two minutes after opening the 532 nm laser shutter. The Fig. 3 shows the evolution towards a full conversion to anatase using only a 75 Wmm^−2^ power density (Fig. 3a,c), and to rutile using a 445 Wmm^−2^ power density (Fig. 3b,d). There, we note that the crystallization process is especially fast due to the higher excitation power and the system can hardly record the start of the conversion.

Raman modes can be sensitive to crystal size. However, this is not case of TiO_2_ for which grain size has no effect on the Raman spectra^76–78^. Defect structures in the TiO_2_ strongly affect the Raman spectrum by producing shifts and broadening of some of the Raman peaks^76^. Among those defects, oxygen vacancies are responsible for the non-stoichiometric effects that cause the shift and broadening of the *Eg* Raman mode^77,78^.

Compared to our conventional (white) crystalline TiO_2_ powder crystallized using thermal annealing (see supplementary section Suppl. Figure S2), we observe a significant shift and broadening of the *E*_*g*_ mode for the red TiO_2_ converted to both anatase and rutile using laser-induced conversion in air. In fact, this *E*_*g*_ mode is known to be more sensitive to oxygen vacancies and it is often used as a direct indicator to detect their presence^79^.

After complete conversion to anatase, Fig. 3c, this dominant *E*_*g*_ mode normally at 156 cm^−1^ and 204 cm^−1^ shifts and broadens significantly compared with conventional (white) TiO_2_ after conventional thermal annealing. After the complete conversion to rutile using higher laser power densities, Fig. 3d shows that the *E*_*g*_ mode normally at 232 cm^−1^ peak also shifts to higher energies (251 cm^−1^). In contrast, the higher vibrational peaks normally at 401, 520 and 643 cm^−1^ for anatase TiO_2_ shift to slightly lower wavenumbers for the red anatase TiO_2_ (396, 511 and 634 cm^−1^ respectively). However, this power-temperature dependence of the anatase Raman signatures is consistent with the literature^80–82^. Similarly, the vibrational peak normally at 451 cm^−1^ for rutile TiO_2_ also shifts to lower wavenumbers for the red rutile TiO_2_ (433 cm^−1^), which is also consistent with the literature^83–85^.

The significant shift and broadening of the *E*_*g*_ mode using laser-induced conversion in air indicates a high concentration of residual oxygen vacancies concentration after crystallization. Indeed, the temperature-dependent color changes, as well as the promotion and the disappearance of oxygen vacancies during the thermal crystallization can be explained using previous models^86^. These models suggest that temperatures between 300 °C and 500 °C promote the entropy-driven outward diffusion of Ti^3+^ defects towards the nanoparticle surface and to produce a black-gray color transition, which is an indicator of the high oxygen vacancy concentration^86^.

**Figure 4:**
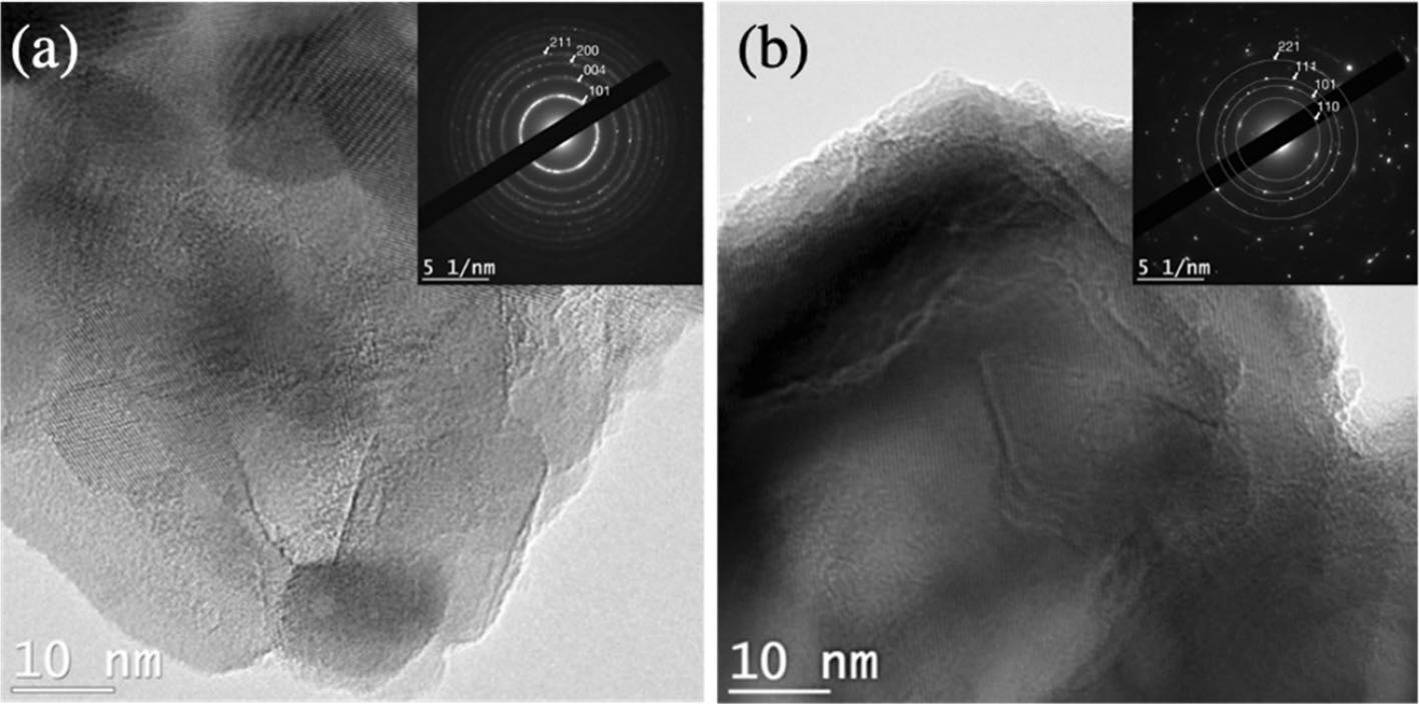
TEM images of red TiO_2_ nanoparticles after laser-induced crystallization. (**a**) Red anatase-TiO_2_ powder. (**b**) Red rutile-TiO_2_ powder.

The lattice structure for laser-converted red TiO_2_ can be directly observed in Fig. 4. All the samples display a well-organized lattice structure and selected-area (electron) diffraction (SAED) analysis confirms the clearly-defined polycrystalline anatase and rutile polymorph structures.

### Thermally-induced crystallization of the oxygen vacancy-rich TiO_2_

While this unique laser-assisted crystallization to anatase or rutile TiO_2_ is only possible for the oxygen vacancy-rich (red) amorphous TiO_2_ powder, conventional thermally-induced crystallization to anatase or rutile TiO_2_ always remains possible. We chose to perform this experiment for both the red and white amorphous TiO_2_ powders shown in Fig. 1a,c to help-us fully understand the complex mechanisms associated with these high oxygen vacancy densities.

**Figure 5:**
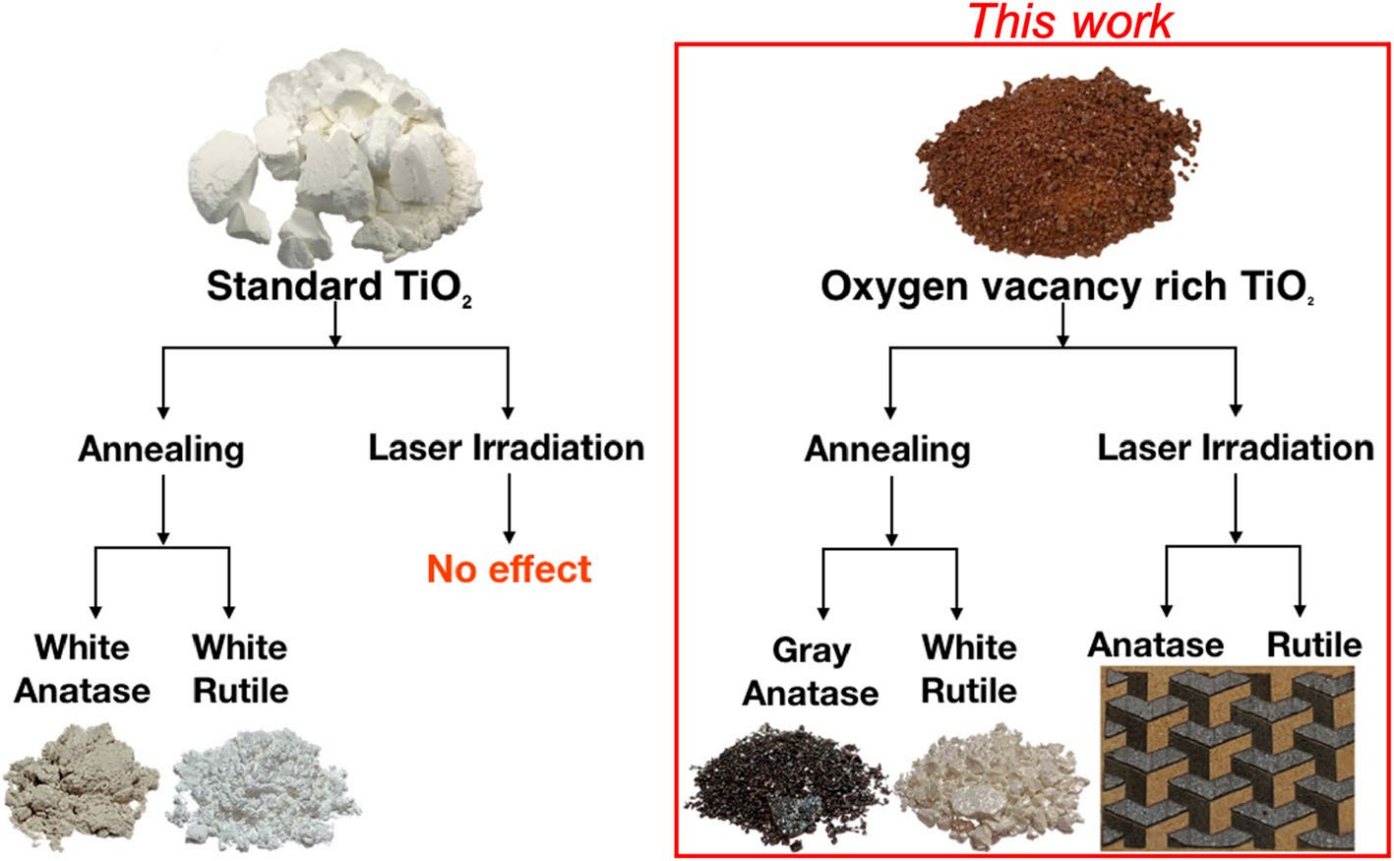
Comparison of the different crystallization processes for the standard (white) and oxygen vacancy-rich (red) amorphous TiO_2_ powders using thermal-annealing and laser-induced crystallization.

As expected, our standard (white) amorphous TiO_2_ powder yields white anatase and white rutile powders after thermal annealing at 450 °C (anatase) and 800 °C (rutile). However, the oxygen vacancy-rich (red) amorphous TiO_2_ powder yields a darker (gray) anatase powder after thermal annealing at 450 °C (anatase) and a white rutile powder after thermal annealing at 800 °C (rutile). Typical examples are shown in Fig. 5.

The grayish color of the anatase is not unusual for this TiO_2_ polymorph^86^. In fact, when anatase TiO_2_ is found in its natural form, it can vary from indigo-blue to black and steely luster^46^. The synthesis of dark anatase has been previously reported by means of UV irradiation followed by annealing under argon atmosphere^87^ or by hydrogenation of anatase powders under high pressure^50^. In both cases the darker color arises as a consequence of the oxygen vacancy-mediated absorption^39,88^. This gray anatase proves better-suited to harvest visible and infrared light, making it more efficient for the photocatalytic reactions^46^. Coupled with platinum, it proves an outstanding material for energy conversion applications including hydrogen generation from water/ethanol solutions^46^. Recently, gray anatase has also been deployed for CO_2_ conversion^89^ and air quality control applications^90^.

**Figure 6:**
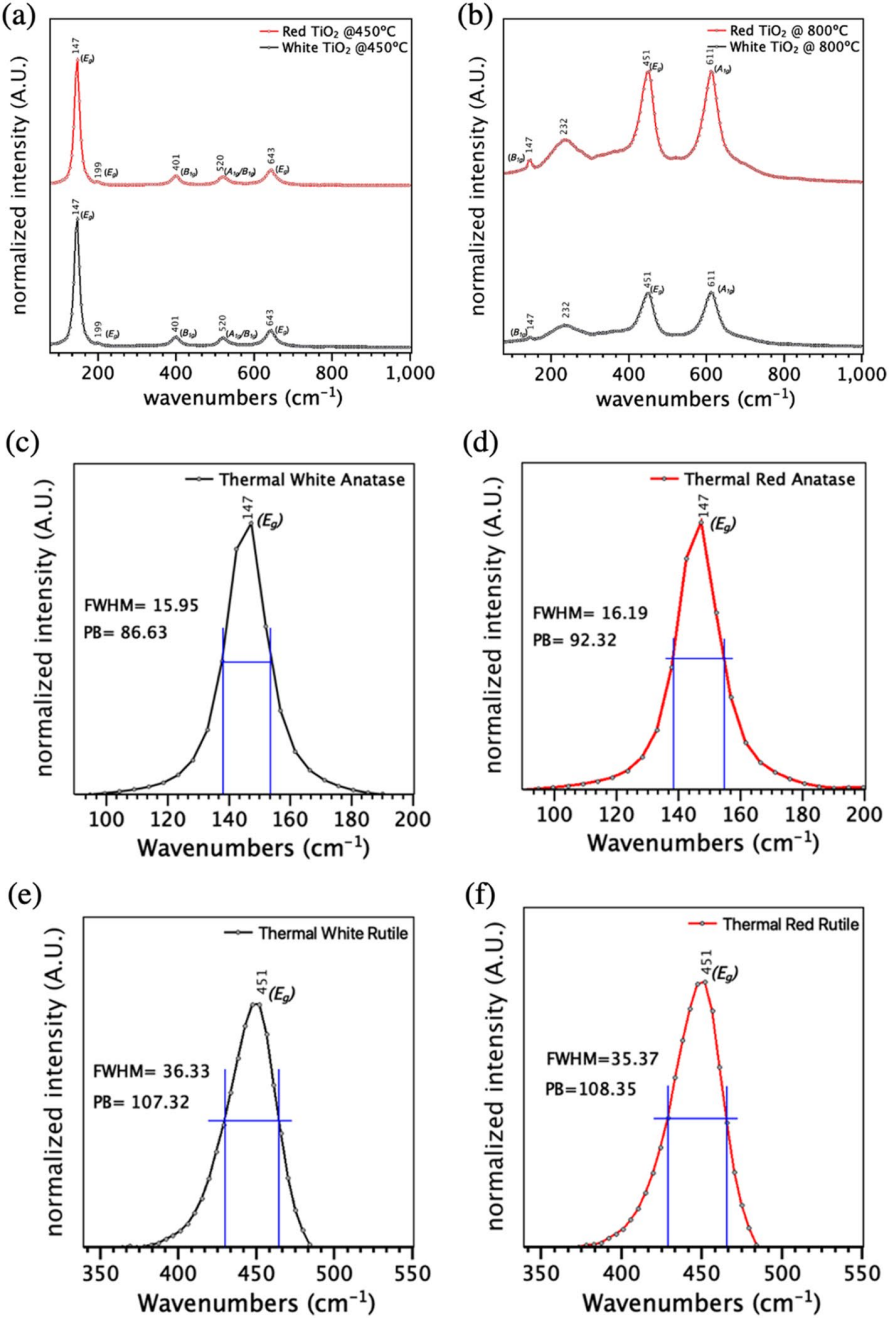
Raman spectra for the different polymorphs crystallized from the amorphous red and white TiO_2_. (**a**) White and red anatase TiO_2_ powders crystallized at 450ºC. (**b**) White and red rutile TiO_2_ powders crystallized at 800ºC. (**c**, **d**) E_g_ mode of the white and red anatase TiO_2_ powders crystallized at 450ºC. (**e**, **f**) E_g_ mode of the white and red rutile TiO_2_ powders crystallized at 800ºC.

Once again, Raman micro-spectroscopy analysis is used to compare the anatase and rutile signatures of the red and white TiO_2_ after the thermal annealing. In Fig. 6a, both the standard (white) and defect-rich (red) amorphous TiO_2_ powders show a well-defined anatase phase with peaks at 147, 199, 401, 520, 634 cm^−1^ after annealing at 450 °C^91^. In contrast, Fig. 6b also shows a well-defined rutile phase with peaks around 147, 232, 451, 611 cm^−1^ for both powders after annealing^92^ at 800 °C.

Once again, the *E*_*g*_ Raman modes for anatase (at 147 cm^−1^) and rutile (at 451 cm^−1^) can be used to compare the crystallized TiO_2_ samples (Fig. 6). After thermal annealing, Fig. 6c,d shows a slight broadening of the *E*_*g*_ peak for the red TiO_2_ due to the oxygen vacancies^93^. For rutile TiO_2_ thermally crystallized at higher temperatures (800 °C), Fig. 6e,f shows no clear distinction between the white and red TiO_2_ after thermal annealing. This suggests near-complete oxygen vacancy removal after thermal annealing. From these results, we can conclude that higher temperatures can also generate enough energy to break the saturated state of Ti^3+^ defects making the gray color fade away significantly decreasing the concentration of oxygen vacancies^86^.

**Figure 7:**
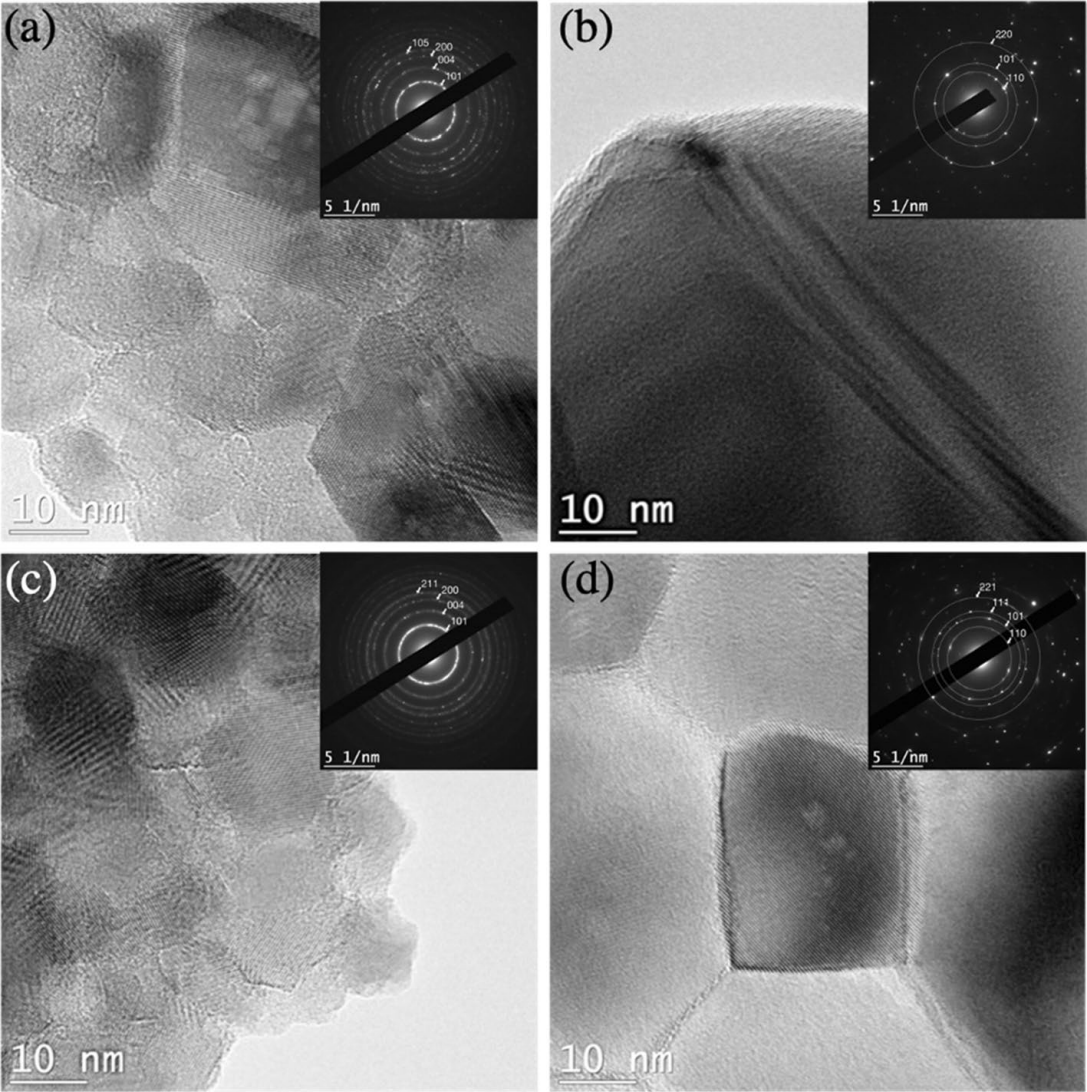
TEM and SAED analysis for the anatase and rutile TiO_2_ powders obtained after thermally-assisted conversion of the standard (white) and defect-rich (red) amorphous TiO_2_ powders. (**a**) Standard (white) anatase TiO_2_ obtained at 450ºC. (**b**) Standard (white) rutile TiO_2_ obtained at 800ºC. (**c**) Defect-rich (red) anatase TiO_2_ obtained at 450ºC. (**d**) Defect-rich (red) rutile TiO_2_ at 800ºC.

The TEM analysis shown in Fig. 7 also confirms the very well-defined crystalline structures for the anatase and rutile TiO_2_ powders obtained after thermally-assisted conversion of the standard (white) and defect-rich (red) amorphous TiO_2_ powders. SAED analysis confirm that all the samples are polycrystalline anatase and rutile polymorphs.

### Laser patterning of complex polymorphic meta-structures using oxygen vacancy-rich TiO_2_

**Figure 8:**
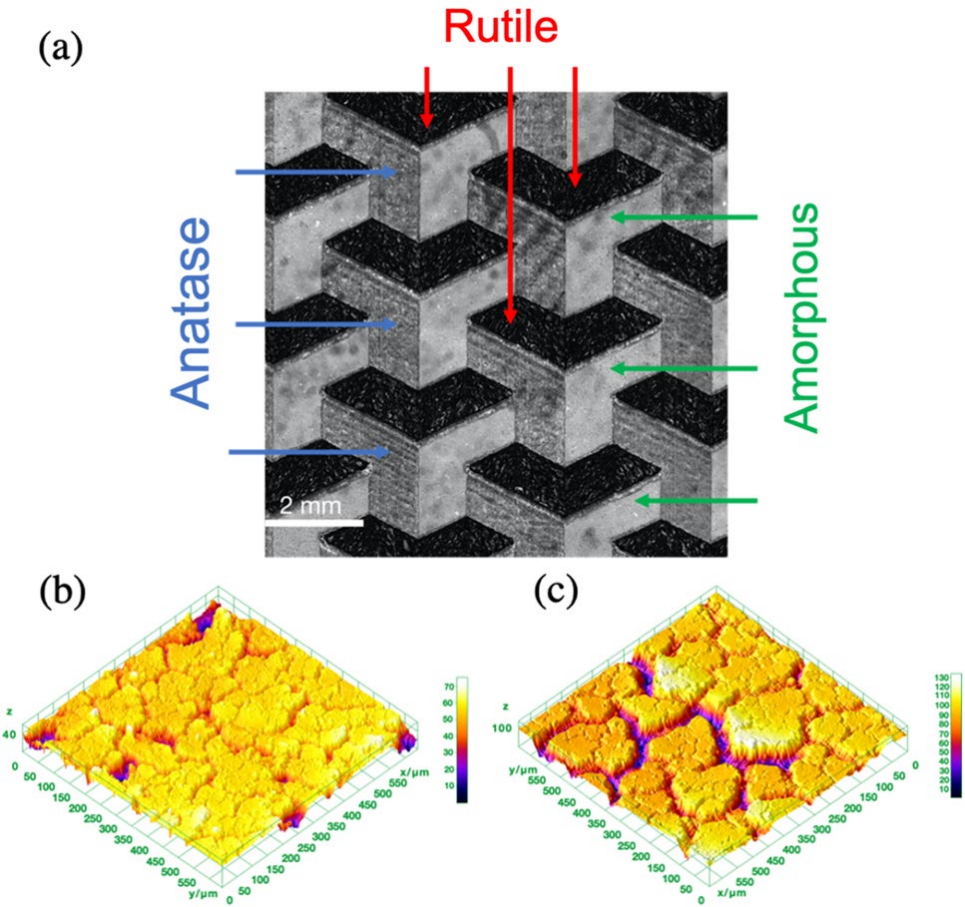
Complex laser crystallized TiO_2_ polymorphic meta-structure. (**a**) Laser scanning microscopy image. (b,c) Typical high-resolution topographic 3D surface reconstructions of the (**b**) anatase and (**c**) rutile areas generated using the software ImageJ based on the data obtained with the laser scanning microscope analysis.

To move one step closer towards the laser-assisted additive ceramic manufacturing, we used a standard commercial filament-based 3D printer mounted with a low-power 405 nm laser printhead. This procedure has been detailed by our team in previous reports^94^. Using this laser, we used 140 W mm^−2^ (for anatase) and 215 W mm^-2^ (for rutile) to form a complex mosaic pattern combining amorphous, anatase and rutile polymorphs originating from the vacancy-rich amorphous TiO_2_^94^. Figure 8 shows it is now possible to spatially organize different crystalline phases with high level of precision within complex architectures and patterns. The procedure and the optimal processing parameters are fully described in the supplementary section.

The laser-converted anatase and rutile areas in Fig. 8 are well-defined and the change in color for each crystallized area matches the results obtained during thermal crystallization of the vacancy-rich (red) amorphous TiO_2_, resulting in dark-grey powder for anatase and white powder as previously described. The structure of the crystallized areas is confirmed by Raman micro-spectroscopy and XRD as shown in the supplementary information (Suppl. Figure S2 and S3 respectively) where the characteristics peaks are clearly identifiable for both polymorphs. The high-resolution topographic 3D surface reconstructions from the converted areas shown Fig. 8b,c are obtained using a laser-scanning microscope (LEXT OLS4100 from Olympus) with ImageJ reconstruction. It reveals cracks in the laser-converted regions, both anatase and rutile. These cracks appear as a direct consequence of the rapid densification of the material during the laser-assisted crystallization process^70^. As expected, the cracks are more pronounced in rutile compared with anatase since its unit-cell volume^95^ is half that of anatase and it possesses a higher density value due to the increased number of atoms^9,10^.

## Conclusion

We report the controlled preparation of oxygen vacancy-rich (*red*) amorphous TiO_2_ nanoparticles by green hydrolytic sol–gel reaction of Ti(OBu)_4_ and using acetylacetone (acac) as a chelating agent, significantly slowing-down the reaction kinetics due to the steric inhibition effect in order to promote oxygen vacancies. FTIR spectroscopy is used to differentiate the new bonds due to the chelating agent and suggest a cyclic dimeric structure for the chelation reaction consistent with experimental models reported in the literature. For comparison, thermally-induced crystallization to anatase and rutile TiO_2_ is performed at 450 °C and 800 °C respectively for our standard non-chelated (white) and the oxygen vacancy-rich (red) amorphous TiO_2_. It yields gray anatase and white rutile after thermal conversion, which is also expected and consistent with the literature. TEM and SAED results show a well-organized highly-crystalline lattice structure for all the samples.

We can exploit these oxygen vacancies to achieve low-energy laser-assisted crystallisation at room-temperature and in ambient environment using this oxygen vacancy-rich (red) amorphous TiO_2_. Using a 532 nm laser with 75 W mm^−2^ and 445 W mm^−2^ power densities yields induces complete crystallization to anatase and rutile, respectively. Transient Raman micro-spectroscopy shows that crystallization occurs within the very first seconds of irradiation, and that this effect is permanent and noncumulative. This laser-induced crystallization is directly attributed to the presence of oxygen vacancies and its reactivity towards molecular oxygen and a process schematic is proposed. Moreover, a standard commercial filament-based 3D printer mounted with a low-power 405 nm laser printhead is used to convert selectively to anatase and rutile. We successfully produced a complex polymorphic mosaic meta-structure combining three (3) TiO_2_ polymorphs with high level of precision to potentially study and exploit new synergistic effects^96,97^. Most importantly, these laser-induced phase transitions are entirely performed at room-temperature in ambient environment, without any kind of dopant in the TiO_2_ prior to photo-activation.

Obviously, these oxygen vacancy-rich nanoparticles strongly absorb visible light. Furthermore, the increase in oxygen vacancies can potentially lead to an increase in the photo-catalysis and energy-harvesting performances of TiO_2_. As it can be easily converted to anatase or rutile TiO_2_ in ambient room conditions using only a low-energy laser excitation, we believe this last breakthrough opens new vistas of possibilities towards the additive manufacturing of engineered crystalline TiO_2_ substrates for photo-catalysis, fuel cells and energy-harvesting applications.

## Materials and methods

In order to prepare the standard (white) amorphous TiO_2_ powder, 28.8532 g of ethanol (Product 1590102500 from Sigma-Aldrich) are mixed with 10.8604 g of titanium(IV) butoxide (Product 244112-500G from Sigma-Aldrich) and this solution is stirred for 40 min. Finally, the hydrolysis reaction is triggered by adding dropwise 0.84 mL of deionized water. Precipitation of the amorphous white TiO_2_ occurs within the first few seconds after the reaction is started. This mixture is aged for 72 h to form the TiO_2_ sol–gel and then the solvent is evaporated at ambient conditions to obtain the white TiO_2_ powder shown in Fig. 1a.

In order to prepare the oxygen-rich (red) amorphous TiO_2_, 28.8532 g of ethanol (Product 1590102500 from Sigma-Aldrich) are mixed with 1.4748 g of acetylacetone (Product P7754-1L-A from Sigma-Aldrich). This solution is stirred for 20 min. Then, 10.8604 g of titanium(IV) butoxide (Product 244112-500G from Sigma-Aldrich) are added and stirred for another 40 min. Finally, the hydrolysis reaction is triggered by adding dropwise 0.84 mL of deionized water. The resulting mixture is stirred for 120 min and then aged for 8 months to form the red TiO_2_ sol–gel. During the aging time, the ethanol slowly evaporates to precipitate a vitreous red TiO_2_. It is important to notice that both syntheses are carried-out entirely at room temperature.

## Supplementary Information

Supplementary Information.

## Data Availability

All data needed to evaluate the conclusions in the paper are present in the paper and/or the supplementary information.
