# Supplementary material for: Synthesis of vacancy-rich titania particles suitable for the additive manufacturing of ceramics
Source: Sci Rep. 2022 Sep 14;12:15441. doi: 10.1038/s41598-022-19824-y (PMC9474447; doi:10.1038/s41598-022-19824-y)
Supplement: Supplementary file 1 — Supplementary Information. [file 41598_2022_19824_MOESM1_ESM.docx]

**Supplementary information for Being perfect is not always better: How vacancy-rich titania particles enable additive ceramic manufacturing**

Jaime A. Benavides, Luis F. Gerlein, Charles Trudeau, Debika Banerjee, Xiaohang Guo, Sylvain G. Cloutier*

*Corresponding author. sylvaing.cloutier@etsmtl.ca


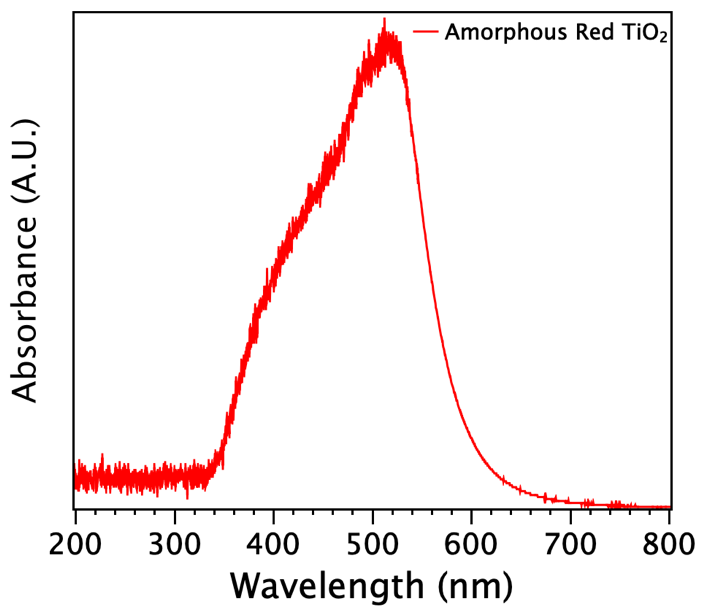


**Fig. S1.**

UV–vis spectra of the amorphous red TiO_2_.

Laser crystallization

In the first demonstration of the laser-assisted conversion, the built-in low-power (60mW) fiber-coupled continuous-wave (CW) laser at 532nm of the WITEC Alpha300 confocal Raman microscope are used for excitation through a low-magnification (10×) objective. Using the built-in variable attenuator, power densities of 75W·mm^−2^ and 445W·mm^−2^ can respectively trigger the anatase and rutile crystallization in our oxygen vacancy-rich (red) amorphous TiO2 at room temperature under ambient conditions.

Laser crystallization using a 3D printer stage

In the second demonstration of the laser-assisted conversion, we use a 405 nm CW laser mounted on a BIBO-2 Touch 3D printer that provides a positional accuracy resolution of 20 µm in the Z direction. The trace of the laser at the focal point is 20µm wide. The pattern created using the software Inkscape is filled with a hatching of contiguous lines 20 um thick. We achieve complete crystallization to rutile by radiating the sample with 215 W mm^-2^, at 300 mm/min of laser speed and 2 passes per section. For anatase crystallization the laser is set to radiate at 140 W mm^-2^, at 450 mm/min of laser speed and 3 passes per section.

*
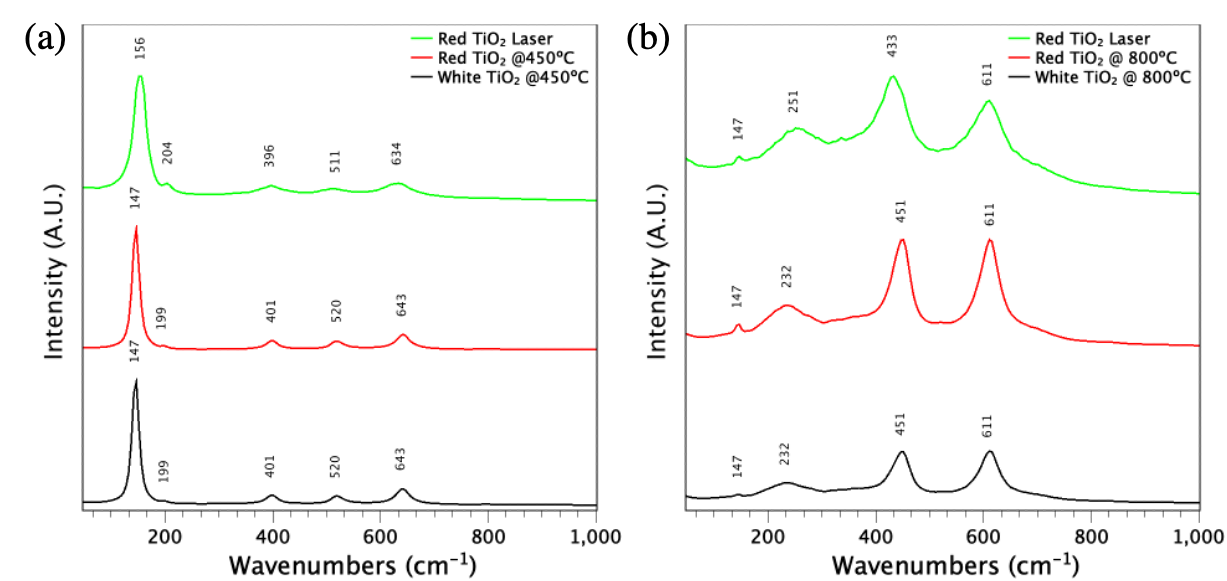
*

**Fig. S2.**

Raman micro-spectroscopy analysis of the (a) anatase and (b) rutile crystallized by laser and thermal methods and the shift in the Raman peaks.


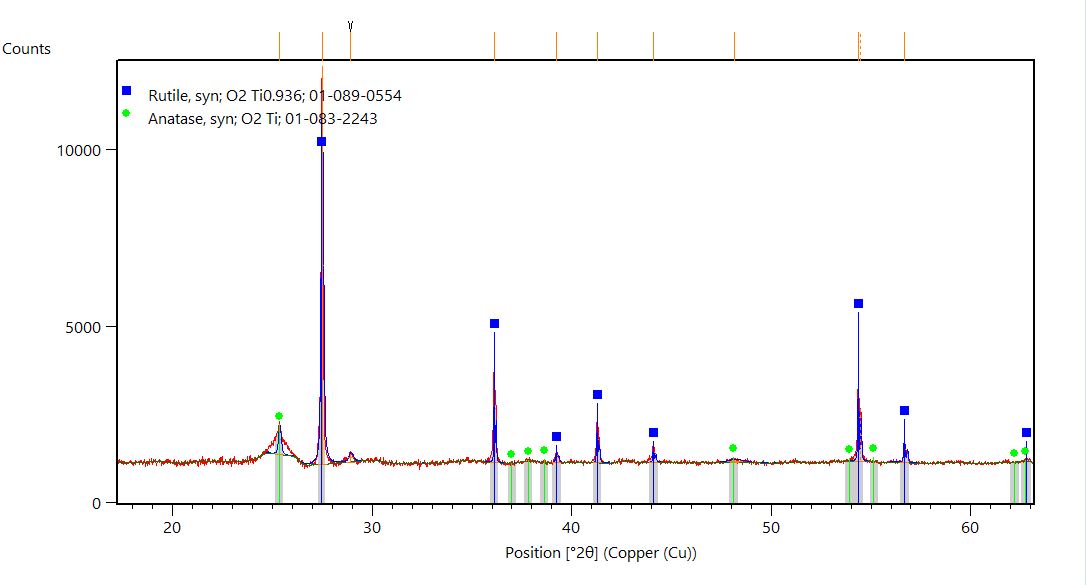


**Fig. S3.**

X-ray diffraction analysis of the anatase and rutile crystallized by laser method.
